# DNA Methylation Signatures Characterize Gene Expression Modulation in Lung Cancer Patients Affected by Anorexia

**DOI:** 10.3390/nu16213721

**Published:** 2024-10-31

**Authors:** Alessio Molfino, Francesca Ambrosani, Silvia Udali, Giovanni Imbimbo, Sara Moruzzi, Annalisa Castagna, Patrizia Pattini, Federica Tambaro, Cesarina Ramaccini, Maurizio Muscaritoli, Simonetta Friso

**Affiliations:** 1 Department of Translational and Precision Medicine, Sapienza University of Rome, 00185 Rome, Italy; giovanni.imbimbo@uniroma1.it (G.I.); federica.tambaro@uniroma1.it (F.T.); cesarina.ramaccini@uniroma1.it (C.R.); maurizio.muscaritoli@uniroma1.it (M.M.); 2 Unit of Internal Medicine B, Department of Medicine, School of Medicine, University of Verona, 37129 Verona, Italy; francesca.ambrosani@univr.it (F.A.); silvia.udali@univr.it (S.U.); sara.moruzzi@univr.it (S.M.); annalisa.castagna@univr.it (A.C.); patrizia.pattini@univr.it (P.P.)

**Keywords:** DNA methylation, NGS, anorexia, lung cancer, appetite, inflammation

## Abstract

Background/Objectives: The pathophysiology of cancer anorexia is multifactorial and unclear. Transcriptomic analysis from PBMCs RNA showed diverse patterns of gene expression pathways in anorexic cancer patients. We assessed whether the different transcriptomic signatures are modulated by DNA methylation in lung cancer patients presenting with poor appetite. Methods: Lung cancer patients and controls were enrolled, and anorexia was assessed by the FAACT-score questionnaire. Genome-wide DNA methylation was determined by Human Infinium MethylationEPIC BeadChip Kit. Data from genome-wide methylation analysis were merged with those from gene expression analysis, previously obtained by RNA sequencing (NGS). Four groups of genes were identified for each comparison: hypermethylated repressed, hypermethylated induced, hypomethylated repressed, and hypomethylated induced. Results: Cancer patients (*n* = 16) showed 382 differentially methylated genes when compared with controls (*n* = 8). Anorexic patients (*n* = 8) presented 586 hypomethylated and 174 hypermethylated genes compared with controls. In anorexic patients vs. non-anorexic (*n* = 8), 211 genes were identified as hypomethylated and 90 hypermethylated. When microarray methylation data were merged with transcriptomic data by RNA sequencing, we observed significant differences in anorexic patients vs. controls; a total of 42 genes resulted as hypomethylated and induced, 5 hypermethylated repressed, 10 hypermethylated induced, and 15 hypomethylated repressed. The CG sites analyzed by targeted bisulfite NGS in four genes of interest (FLNA, PGRMC1, GNL3L, and FHL1) resulting as hypomethylated in anorexic vs. controls allowed the validation of the data obtained from DNA methylation. Interestingly, the four genes resulted as hypomethylated in anorexic patients vs. non-anorexic patients and vs. controls (*p* < 0.0001). Conclusions: Our data support that methylation is implicated in cancer-associated anorexia and nutritional derangements among lung cancer patients.

## 1. Introduction

Appetite dysregulation is highly prevalent in cancer patients and is associated with the development of body weight loss and cachexia [1,2,3]. Poor appetite, known as anorexia, is often related to negative outcomes in cancer, including reduced response to therapies, poor quality of life, and higher mortality rate [4,5]. There are several pathophysiological mechanisms implicated in the development of anorexia in cancer, although most of them are still under investigation and are not completely clarified.

Experimental evidence indicates that inflammation and altered immune functions are amplified within the central nervous system (CNS), sustaining the activity of neuronal populations, negatively regulating appetite, and also affecting proteolysis and lipolysis in the periphery [6,7,8]. Among these complex phenomena, we recently showed that changes in gene expression are present in patients with cancer according to the presence of anorexia [9]. In fact, we described a modulation in the expression of different genes associated with low appetite that may be associated with altered energy homeostasis and leading to malnutrition and cachexia in lung cancer [9]. In particular, the main pathways involved in appetite dysregulation were those related to inflammatory status and cytokine and chemokine pathways [9]. In addition, in the frame of gene expression regulation, it is highly plausible that specific epigenetic signatures may be present during cancer anorexia. In fact, in other catabolic conditions, mainly characterized by muscle loss, a role for epigenetic control was hypothesized, including changes in gene-specific DNA methylation [10].

Epigenetic phenomena, in particular DNA methylation, are mechanisms of gene expression regulation that can be influenced by environmental exposure [11] and nutritional status [12] and that have been extensively studied in cancer [13,14].

These conditions are highly tissue-specific, and the study of DNA methylation in cancer tissues allowed the identification of possible pathogenetic mechanisms in different cancer types [13,15,16].

Another functional approach is to identify specific epigenetic biomarkers for cancer in the blood, an easily accessible tissue, both for early cancer diagnosis and for prognostic purposes. The idea is that the methylation status of blood cells, in particular peripheral blood mononuclear cells (PBMCs), could reflect systemic phenomena, and this aspect is a matter of scientific debate [17].

In the present study, we aimed to assess DNA methylation and gene expression patterns in PBMCs obtained from lung cancer patients in order to investigate the possible role of epigenetics, specifically in the setting of human cancer anorexia.

## 2. Materials and Methods

### 2.1. Patients and Control Group Selection

Patients with a new diagnosis of lung cancer who did not receive any anticancer treatment, including chemo- or immunotherapy, radiotherapy, or surgery, were enrolled at the Department of Translational and Precision Medicine, Sapienza University of Rome, Azienda Policlinico Umberto I, Rome, Italy. As previously described [9], inclusion criteria were age ≥ 18 years and the ability to sign the informed consent to this study. The selected participants were those included in the previous study analyzing gene expression in cancer anorexia [9]. We excluded patients with other forms of wasting conditions different from lung cancer, those with neurological impairments, and those with other catabolic disorders, including liver failure, chronic kidney disease, or others. The control group included subjects with no history of cancer or other catabolic diseases enrolled at the same study site. This study was performed according to the Declaration of Helsinki and approved by the local Ethics Committee (Sapienza University of Rome, Italy Azienda Policlinico Umberto I—prot. N. 1162/17, 15 December 2017).

### 2.2. Nutritional, Clinical Parameters and Appetite Evaluation

All the participants were evaluated in terms of body weight, height, body mass index (BMI), presence or absence of body weight loss in the previous six months, and serum levels of C-reactive protein as a marker of inflammatory status, with standard technique.

As previously described [9] and validated [1], regarding the assessment of appetite, we used the Functional Assessment of Anorexia/Cachexia Therapy (FAACT) score, and a value of ≤30 allowed us to make the diagnosis of anorexia [1].

During the study visit, demographic data, patients’ clinical history, and information regarding the presence of comorbidities were collected.

### 2.3. Blood Samples Collection and DNA Extraction from PBMCs

For each study subject, peripheral blood samples were drawn in an EDTA Vacutainer and centrifuged at 2500× *g* at Room Temperature (RT) for 15 min. The buffy coat was collected, and the PBMCs were isolated by density gradient centrifugation using Ficoll (Ficoll-Paque™ plus, GE Healthcare, Chicago, IL, USA), according to manufacturer protocol.

DNA was extracted from PBMCs using AllPrep DNA/RNA/Protein Mini kit (Qiagen, Hilden, Germany) following manufacturer instructions and quantified using Qubit 4 Fluorometer (Thermo Fisher Scientific, Wilmington, DE, USA). DNA purity was assessed by NanoDrop1000 spectrophotometer (Thermo Fisher Scientific, Wilmington, DE, USA), and DNA integrity was evaluated by gel electrophoresis with 1.5% agarose.

### 2.4. Genome-Wide DNA Methylation Analysis

Genome-wide DNA methylation analysis was performed with Human Infinium MethylationEPIC BeadChip Kit (Illumina, San Diego, CA, USA) that allows covering more than 850,000 methylation sites per sample, including promoters, CpG islands, gene bodies, and enhancer regions. In particular, the target regions include RefSeq genes, transcription factor binding sites, and enhancer regions identified by the ENCODE project and FANTOM5, as reported by the Illumina datasheet.

The analysis was carried out on the participants to compare the DNA methylation status of anorexic cancer patients, non-anorexic cancer patients, and healthy controls.

In brief, 250 ng DNA was bisulfite converted and whole-genome amplified for 20 h, followed by end-point fragmentation. Fragmented DNA was precipitated, denatured, and hybridized to the BeadChips for 20 h at 48 °C. The BeadChips were washed, and the hybridized primers were extended with labeled nucleotides showing different signals according to the methylation status of the CG site. The BeadChips were then scanned using the Illumina iScan system, and GenomeStudio software V-1-0-b5 was used for the extraction of DNA methylation signals from scanned arrays. Hypermethylated CpGs were selected with a cut-off of delta beta > 0.2 and hypomethylated CpGs with a delta beta < −0.2 and a *p*-value < 0.05 in each comparison (anorexic cancer patients versus controls; non-anorexic cancer patients versus controls; cancer patients versus controls anorexic; cancer patients versus non-anorexic cancer patient).

Differentially methylated genes were considered those with at least one CpG either hypermethylated or hypomethylated, and enrichment analysis was performed by Enrichr (https://maayanlab.cloud/Enrichr/ (accessed in January 2023)) and PANTHER (Protein ANalysis THrough Evolutionary Relationships) classification system (http://www.pantherdb.org (accessed in January 2023)) to cluster genes according to metabolic and cell signaling pathways.

### 2.5. Merging of DNA Methylation and Gene Expression Data

Data from genome-wide methylation analysis were merged with those from gene expression analysis, previously obtained with RNA Sequencing [9].

Differentially expressed genes were defined considering a threshold of log2FoldChange > 0.58 and a *p*-value < 0.01 for induced genes and a threshold of log2FoldChange < −0.58 and a *p*-value < 0.01 for repressed genes. Four groups of genes were identified in each comparison: hypermethylated repressed, hypermethylated induced, hypomethylated repressed, and hypomethylated induced genes.

### 2.6. Targeted Bisulfite Sequencing

Microarray DNA methylation data were then validated using targeted bisulfite Next-Generation Sequencing (NGS) methodology.

Four hypomethylated induced genes in anorexic cancer patients compared with healthy controls were selected to validate their methylation status (FLNA, PGRMC1, GNL3L, and FHL1). Primer pairs were designed to amplify specific CpG sites for each gene (Supplementary Table S1). The samples were bisulfite converted using the EZ DNA Methylation-Lightning™ Kit (Zymo Research, Irvine, CA, USA) according to the manufacturer’s instructions, and the regions of interest were amplified using specific primer pairs (Supplementary Table S1). The resulting amplicons were pooled for harvesting and subsequent barcoding using limited amplification cycles. After barcoding, samples were pooled, purified (DNA Clean & Concentrator-5™, Zymo Research, Irvine, CA, USA), and prepared for massively parallel sequencing using a MiSeq V2 300 bp Reagent Kit (Illumina, San Diego, CA, USA) and paired-end sequencing protocol according to the manufacturer’s guidelines. Illumina base-calling was used to analyze the identified reads, then analyzed with Zymo Research proprietary analysis pipeline. Low-quality nucleotides and adapter sequences were trimmed off during analysis Quality Control. Sequence reads were aligned back to the reference genome using Bismark (http://www.bioinformatics.babraham.ac.uk/projects/bismark/ (accessed in October 2022)), and paired-end alignment was used as default requiring both reads to be aligned within a certain distance; otherwise, both were discarded. Index files were constructed using the bismark_genome_preparation command and the entire reference genome. The non_directional parameter was applied while running Bismark. All other parameters were set to default. Nucleotides in primers were trimmed off from amplicons during methylation calling. The methylation level of each sample cytosine was estimated as the number of reads reporting a C, divided by the total number of reads reporting a C or T. Data obtained were analyzed by performing the Fisher test, setting a cutoff *p*-value < 0.05 for statistically significative genes and a methylation difference <−0.1 or >0.1 to define the genes, respectively, hypomethylated and hypermethylated.

### 2.7. Statistical Analyses

The data are shown as the mean ± standard deviation and median with 25th and 75th percentiles for continuous normally and non-normally distributed variables, as appropriate. Normal distribution was tested by the Shapiro–Wilk test. Variables as categories are shown as numbers (%). We verified differences among groups (patients with or without anorexia, or controls) by Analysis of Variance (ANOVA) and by the Kruskal–Wallis test, as appropriate. We then used the two-tailed t-test or Mann–Whitney test according to normality/non-normality to analyze the statistical significance.

Considering the absence of data on DNA methylation in this specific clinical setting (cancer-associated anorexia), we performed a post hoc power analysis. Given an alpha error of 5%, an allocation ratio of 1:1, a sample size of each group of 8 participants (anorexic, non-anorexic, and controls), and mean methylation values of the following genes: GNL3L, FHL1, FLNA, and PGRMC, the “Post hoc: Compute achieved power” procedure (GPower 3.1.9.7) allowed to obtain power of at least 95% for all the four genes.

A *p*-value < 0.05 was set as significant, whereas a *p*-value < 0.01 was considered highly significant. The statistical analysis was performed using IBM^®^ SPSS Statistics version 26.0 (SPSS Inc., Chicago, IL, USA) and the experiments were performed at least three times.

## 3. Results

### 3.1. Study Participant Characteristics

The lung cancer patients and healthy controls included in these analyses were 16 (13 men) and 8 (3 men), respectively. In the cancer group, anorexia was present in 50% of the sample (*n* = 8), and none of the controls had appetite impairments. The main clinical characteristics of all the participants are reported in Table 1. In summary, cancer patients presented with a mean age of 65.3 ± 11.6 years, BMI of 24.8 ± 4.1 kg/m^2^, a median body weight loss in the previous 6 months of 5.7% (1.2; 8.1), and a mean FAACT score of 31.2 ± 7.7. Fourteen patients (88%) had stage 4 of the disease (Table 1). Patients with cancer anorexia were male (100%) and presented with higher median body weight loss (%) with respect to those without anorexia (7.8, 5.4; 10.7 vs. 2.3, 0.0; 6.8, *p* = 0.038).

The control group included subjects working at the Department of Translational and Precision Medicine and outpatients with no history of cancer or other catabolic diseases. The mean age of this group was 56.4 ± 11.6 years, and BMI was 25.9 ± 5.6 kg/m^2^.

No involuntary body weight loss was present in the prior 6 months (Table 1).

### 3.2. DNA Methylation and Gene Expression Patterns in PBMCs of Lung Cancer Patients

Methylation analysis by microarrays technology allowed us to identify differentially methylated genes in cancer patients as compared with healthy controls, in particular 282 hypomethylated and 100 hypermethylated genes (Table 2).

As shown in Table 2, we also performed other comparisons, i.e., anorexic cancer patients vs. healthy controls, non-anorexic cancer patients vs. healthy controls, and anorexic cancer patients vs. non-anorexic cancer patients. Most of the differences in terms of differentially methylated genes were found when comparing anorexic cancer patients with controls (586 hypomethylated and 174 hypermethylated genes).

**Figure 1:**
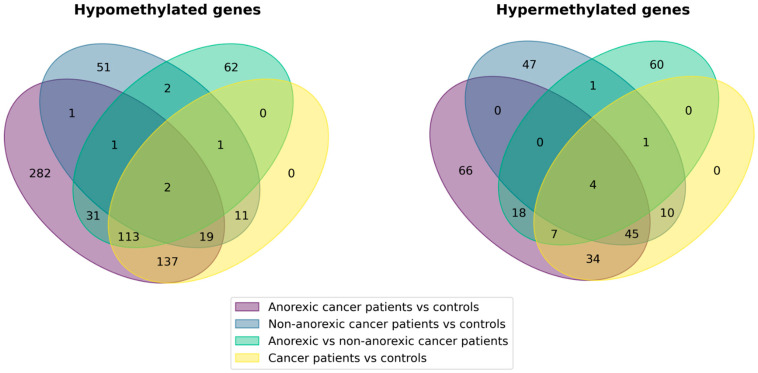
Venn diagrams representing the differentially methylated genes, either hypomethylated or hypermethylated for the comparison between each group as indicated in the legend.

Venn diagrams representing the number of hypomethylated or hypermethylated genes in the four comparisons are reported in Figure 1.

**Figure 2:**
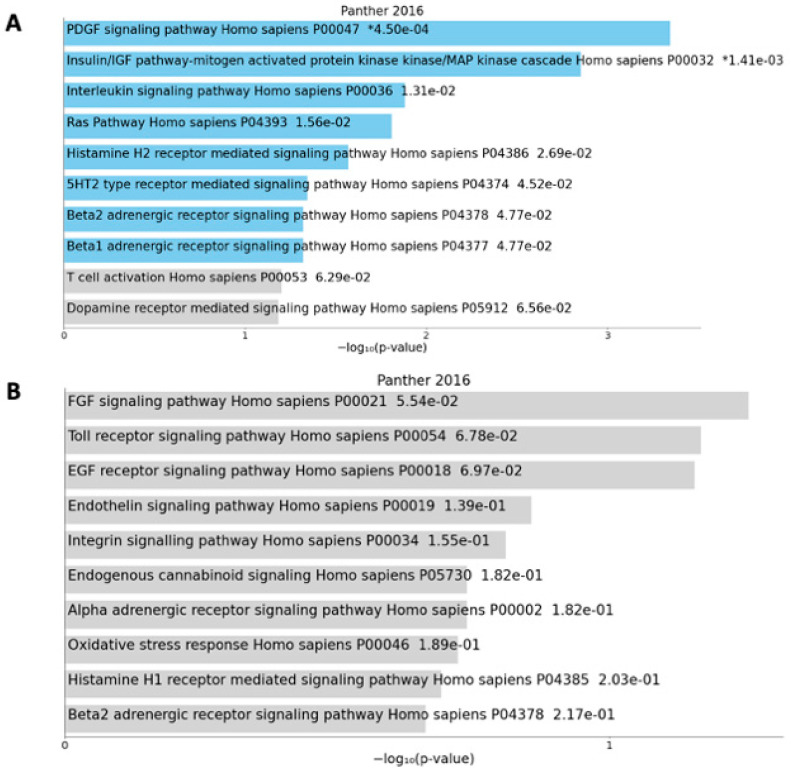
Enriched pathways in the hypomethylated (**A**) and hypermethylated (**B**) genes in the comparison between anorexic cancer patients and healthy controls. The bar chart shows the top 10 enriched terms according to Panther classification, along with the corresponding *p*-values. Blue bars correspond to terms with significant *p*-values (<0.05). An asterisk (*) next to the *p*-value indicates the term also has a significant adjusted *p*-value (<0.05).

The differentially methylated genes that characterized anorexic cancer patients compared with controls were then classified with the Panther classification system to identify the main enriched pathways represented (Figure 2).

As shown in Figure 2, several hypomethylated genes (Panel A) were involved in signaling pathways whose dysregulation is related to carcinogenesis and cancer proliferation (Insulin/IGF pathway–mitogen-activated protein kinase /MAP kinase cascade, PDGF signaling pathway, Ras Pathway), while others were involved in immune and inflammatory response (Interleukin signaling pathway, Histamine H2 receptor-mediated signaling pathway, T cell activation). In Figure 2, Panel B, the pathways identified in the group of hypermethylated genes are reported; however, they were not statistically significant. Among them, it was possible to observe genes involved in the regulation of cellular processes, such as cell survival, proliferation, migration, and differentiation (FGF signaling pathway, EGF receptor signaling pathway, Endothelin signaling pathway, Integrin signaling pathway), and in the immune response (Toll receptor signaling pathway, Histamine H1 receptor-mediated signaling pathway).

The complete lists of hypomethylated and hypermethylated genes of the different pathways are reported in Supplementary Tables S3 and S4.

Moreover, when anorexic patients were compared with non-anorexic patients, 211 genes resulted in hypomethylated and 90 hypermethylated (Table 2).

Microarray methylation data were then merged with previously obtained RNA-sequencing data to identify differentially methylated and differentially expressed genes.

For each comparison, four groups of genes were identified: hypomethylated induced, hypomethylated repressed, hypermethylated induced, and hypermethylated repressed. The genes for which the role of DNA methylation appeared the most plausible in regulating gene expression, according to well-recognized knowledge, were those that resulted in hypermethylated repressed and hypomethylated induced.

In Table 3, we reported the lists of genes that were differentially expressed and methylated within the four comparison settings. We focused our attention on the comparison between anorexic cancer patients and healthy controls, where 42 genes resulted in hypomethylated induced, 5 hypermethylated repressed, 10 hypermethylated induced, and 15 hypomethylated repressed.

Among the 42 hypomethylated induced genes, 10 (ANK1, ARHGAP6, ARHGEF12, FLNA, MSN, SHROOM4, TMSB4X, TMSB4Y, and TNS1) were involved in the regulation of actin cytoskeleton and cell migration; 9 genes (FAM50A, FMR1, GNL3L, HTATSF1, RPGR, THOC2, TMSBAX, TRNS1, and UPF3B) in RNA binding; 6 genes (CHD7, HMGN5, NKAP, POLA1, TRIM26, and VDR) in DNA binding; 5 genes (MPP1, OPHN1, REPS2, TIMP1, and WDR13) in signal transduction; and 3 genes (FHL1, LONRF3, and PGRMC1) in ion channel and metal binding.

Five genes were hypermethylated repressed: FAM53B is involved in the regulation of the Wnt signaling pathway; LSM5 plays a role in pre-mRNA splicing; MOV10 shows RNA binding and helicase activity; SUPT20H is predicted to be involved in the regulation of transcription, and XIST is a non-protein coding gene involved in chromosome X inactivation.

### 3.3. Targeted Bisulfite Sequencing of Four Genes of Interest

Four genes of interest (GNL3L, FHL1, FLNA, and PGRMC1) were selected based on the greatest differential methylation status level among the groups. The four genes resulted in hypomethylated in anorexic patients when compared with healthy controls and significantly upregulated (GNL3L log2FoldChange = 1.11, FHL1 log2FoldChange = 1.03, FLNA log2FoldChange = 0.65, and PGRMC1 log2FoldChange = 1.41). These genes were selected to validate methylation data by targeted bisulfite sequencing and to deeply investigate the promoter methylation status at single CGs level. Indeed, this approach allowed us to assess the methylation status of the cytosines previously analyzed by array-based technique and to evaluate the methylation levels of other CGs present in the sequenced amplicon region.

**Figure 3:**
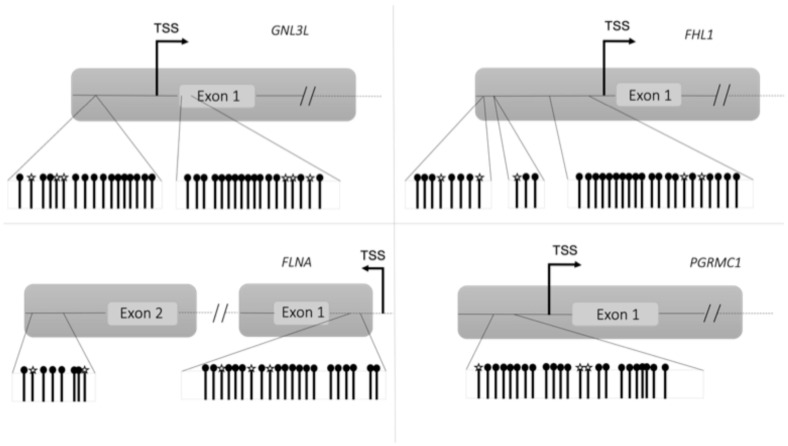
Schematic representation of amplicon regions and hypomethylated CGs in the 4 genes analyzed by targeted bisulfite sequencing. 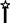 CGs detected by microarray and validated by targeted bisulfite sequencing analysis. 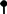 CGs detected with targeted bisulfite sequencing.

In Figure 3, the amplicon regions are represented, as well as the CG sites detected by bisulfite sequencing in the four genes. 

The CG sites analyzed by targeted bisulfite sequencing in the four genes resulted in hypomethylated in anorexic patients when compared with healthy controls, validating the data obtained from methylation analysis by microarray.

In Supplementary Table S2, the methylation values of each CG that resulted in hypomethylated in anorexic cancer patients compared with healthy controls in both approaches using as reference genome Human GRCH37/hg19 are represented. In microarray analysis, a delta beta < −0.2 was set as a cut-off to consider a hypomethylated CG, while a methylation difference < −0.1 was set as a threshold in targeted bisulfite sequencing. In both approaches, the selected CGs resulted in significantly hypomethylated in anorexic cancer patients compared with controls.

The methylation status of the four genes of interest, considering all the CGs assessed by targeted bisulfite sequencing, was then considered in the three study groups, i.e., anorexic cancer patients, non-anorexic cancer patients, and controls.

**Figure 4:**
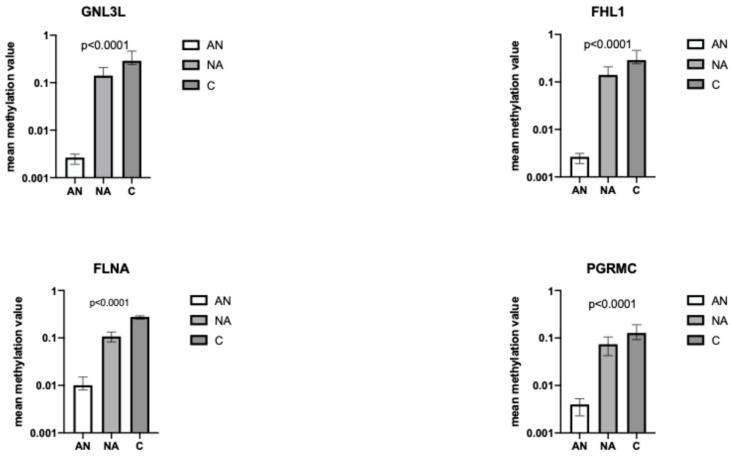
Differences in mean methylation value between anorexic (AN), non-anorexic (NA), and controls of the following genes: GNL3L, FHL1, FLNA, and PGRMC. Graphed: Median value + 95% CI.

In Figure 4, the mean methylation values for each gene comparing the three different groups are reported. The median methylation values were calculated on 34 CG sites for GNL3L, on 32 CGs for FHL1, on 30 CGs for FLNA, and on 26 CGs for PGRMC1.

The four genes showed a promoter DNA methylation status progressively decreasing across the three groups, i.e., healthy controls, non-anorexic, and anorexic cancer patients (*p* < 0.0001) (Figure 4).

## 4. Discussion

This is the first study in humans providing information on DNA methylation changes associated with the presence of cancer anorexia. In particular, all the patients enrolled did not receive any anticancer treatment, therefore excluding any possible inference of therapies with methylation status.

Methylation from blood-derived DNA may be a useful informative tool for the prediction of multifactorial diseases [18], including cancer [19], as we also previously demonstrated by showing that genomic DNA methylation in the PBMCs may be a useful epigenetic biomarker for early detection and cancer risk estimation, independently of the type of cancer [20], and that it could be a prognostic biomarker in other cancers, including hepatocellular carcinoma and cholangiocarcinoma [21].

In the present study, genome-wide methylation data showed the greatest differences in the comparison between anorexic cancer patients and controls, suggesting a possible role of anorexia per se in modifying DNA methylation patterns in PBMCs. As expected, several differentially methylated genes were involved in carcinogenesis and inflammatory response [6,9]. Based on these observations, we believe that the epigenetic mechanisms of DNA methylation may affect specific pathways, including those involved in inflammation, in patients with cancer, possibly promoting alterations in nutritional status (e.g., anorexia). However, due to the exploratory nature of our study, this hypothesis should be confirmed, at least in experimental models aimed at mechanistically evaluating the role of epigenetic modulations in the development of cancer-associated anorexia.

Moreover, in the same comparison, when DNA methylation data were merged with previously obtained gene expression data [9], we identified 42 hypomethylated induced genes. Some of them are known to be implicated in the regulation of actin cytoskeleton and cell migration, and others in RNA and DNA binding; five genes play a role in signal transduction, and three genes, including FHL1, LONRF3, and PGRMC1, in ion channel and metal binding. On the other side, the genes hypermethylated repressed are involved, among others, in signal transduction pathways, as well as in pre-mRNA splicing, RNA binding, and helicase activity. Therefore, these genes are involved in many complex cell functions at different levels in cancer [22]. Our results suggest that an epigenetic signature may be present in cancer-related anorexia, although we observed changes in the methylation status of unspecific genes with several functions not exclusively involved in appetite regulation and metabolism.

Moreover, targeted bisulfite NGS methodology validated microarray data on four selected genes (GNL3L, FHL1, FLNA, and PGRMC1), confirming that they were hypomethylated in patients with cancer anorexia in comparison with controls. Interestingly, a trend for DNA methylation decrease was observed in the three experimental groups, suggesting a strong association between the hypomethylation of these genes and anorexia, more than with cancer disease itself.

Among the genes of interest, FHL1 is a gene known to be associated with changes in cell proliferation, in particular in cancer progression, by suppressing tumor cell growth [23]. Gain- and loss-function studies found that FHL1 inhibits cancer cell growth, migration, and invasion [24]. Also, FHL1 gene silencing was associated with cell proliferation and invasion in human bladder cancer cells [25]. In addition, FHL1 plays a relevant role in muscle tissue development and is implicated in some myopathies. Therefore, we cannot exclude a role for FHL1 in the development not only of anorexia in cancer but likely in muscle derangement frequently observed when developing a catabolic status.

Interestingly, our results indicate the presence of hypomethylation of FNLA, which has also been recently described to play a crucial role in the development of sarcopenia in older adults [26].

In fact, Furutani et al. described FNLA as a potential novel biomarker of muscle loss in geriatric conditions by using a combination of different clinical information and RNA sequencing analysis obtained from PBMCs [26].

By our analyses, in patients with cancer anorexia, we also observed a hypomethylation of GNL3L. This gene codifies a recently identified GTP-binding nucleolar protein that belongs to the HSR1-MMR1 subfamily of GTPases [27]. It is involved in cell growth and proliferation and has been implicated in tumor promotion by stabilizing MDM2 and influencing the progression of cell division [28]. This gene is differentially expressed in many cancer types. From the results of the overexpression and knockdown studies, authors suggested that GNL3L acts in particular on the proliferation of esophageal cancer cells [29]. The data reported also highlight the role of GNL3L in terms of clinical value and prognosis, supporting its role as a novel pan-cancer biomarker [29]. Regarding this specific aspect, especially when developing anorexia in cancer—as in our sample of lung cancer patients—the use of a novel biomarker may be clinically useful in stratifying cancer risk and possibly ameliorating some outcomes.

Importantly, during cancer and specifically in cancer anorexia, several metabolic and hormonal changes can be present and are deeply involved in the complex pathophysiology of poor appetite associated with cancer disease and the consequent catabolic status [6]. Our data indicate a hypomethylation of PGRMC1 in patients with anorexia; however, this pathway is not clearly known to be involved in this condition. Experimental studies indicate that PGRMC1 was frequently elevated during cancer and is a gene involved in many metabolic pathways, including lipid metabolism as well as autophagy and cell proliferation, migration, and invasion [30]. Interestingly, clinical evidence indicates that the expressions of autophagic markers, including LC3B and PINK1 mRNA levels, were modulated among cancer patients with anorexia and cachexia [31].

Our study has limitations, including the type of population studied that was represented by lung cancer patients in an advanced stage of the disease, although the patients were all naïve to any anticancer therapies; the majority of cancer patients were men, and the methylation analysis was performed only on PBMCs and not on other tissues (e.g., lung cancer), although PBMCs were highly reliable to study metabolic and nutritional changes during cancer, including anorexia [9,32,33]. Some genes are also involved in sarcopenia, and we did not evaluate muscularity in our cohort because we focused specifically on anorexia status. Cancer itself, independent of the presence of anorexia, may be the cause of some of the changes in methylation status we observed. All patients with anorexia in our cohort were male, and further investigation is also necessary in female patients.

## 5. Conclusions

Our study provides evidence of changes in DNA methylation patterns during cancer anorexia. This finding highlights the potential relevance of DNA methylation as a biomarker of poor appetite in cancer, and for this reason, the methylation level could, in the future, be analyzed in the diagnosis and follow-up processes if further confirmed. Moreover, our findings indicate that targeting DNA methylation may represent an intriguing field for novel therapeutic interventions in this setting. In fact, in recent years, advancement in the field of epigenetic modulation has largely accelerated, with epigenomic programming representing the most updated one [34]. Therefore, a new scenario for the treatment of cancer anorexia may be represented by novel drugs modulating the methylation status. Further research has the potential to unravel the underlying mechanisms connecting anorexia, cancer, and epigenetic modulation, ultimately leading to an improved and deeper understanding of the intricate interplay between these disorders/phenomena.

## Figures and Tables

**Table 1:** Study participant characteristics.

Parameter	Patients with Lung Cancer	Controls
	(*n* = 16)	(*n* = 8)
Males, *n* (%)	13 (81)	3 (38) *
Age, y	65 ± 12	56 ± 13
BMI, kg/m^2^	24.8 ± 4.1	26.0 ± 5.6
Body weight loss in the previous six months, %	5.7 (1.1; 8.1)	0 (0; 0) ^#^
Hemoglobin, g/dL	13.1 ± 2.1	13.4 ± 2.7
Albumin, g/dL	3.7 ± 0.6	3.9 ± 0.7
C-reactive protein, mg/dL	5.1 (1; 7.5)	0.28 (0.13; 1.05) ^§^
Anorexia according to FAACT score ≤ 30	8 (50%)	/
*Main comorbidities*		
Hypertension, *n* (%)	3 (19)	2 (25)
Diabetes, *n* (%)	1 (6)	1 (13)
Dyslipidemia, *n* (%)	2 (13)	2 (25)

Differences between patients with lung cancer and controls: * *p* = 0.03; ^#^ *p* = 0.004; ^§^ *p* = 0.005.

**Table 2:** Numbers of differentially methylated genes in each comparison.

	Hypomethylated Genes	Hypermethylated Genes
Cancer patients vs. controls	282	100
Anorexic cancer patients vs. controls	586	174
Non-anorexic cancer patients vs. controls	87	107
Anorexic vs. non-anorexic cancer patients	211	90

**Table 3:** List of differentially methylated genes obtained with genome-wide methylation analysis.

	Hypermethylated Genes		Hypomethylated Genes		
	Induced	Repressed	Induced		Repressed
Cancer patients vs. controls	*ATL1* *FAM50A* *FHL1* *GNL3L* *LONRF3* *MPP1* *MSN* *NSDHL* *OPHN1* *PCYT1B* *PGRMC1* *POLA1* *SAT1* *SLC38A5* *THOC2* *UPF3B* *WDR44* *XK*	*NFATC1* *NUP93* *OGT*	*ARHGAP30* *CDK2AP1* *MAP2K3* *NLGN4Y* *NSRP1* *PCSK6* *RYR2* *SLC1A3* *TSPAN9*		
Anorexic cancer patients vs. controls	*ANKRD33B* *ARHGAP30* *GNAS* *MAP2K3* *NSRP1* *PACSIN2* *RYR2* *SRPK2* *TMSB4Y* *TSPAN9*	*FAM53B* *LSM5* *MOV10* *SUPT20H* *XIST*	*ANK1* *ARHGAP6* *ARHGEF12* *CHD7* *FAM50A* *FHL1* *FLNA* *FMR1* *GLA* *GNL3L* *HMGN5* *HTATSF1* *KIF4A* *LANCL3* *LONRF3* *MPP1* *MSN* *NKAP* *OPHN1* *PCYT1B* *PGRMC1*	*POLA1* *REPS2* *RPGR* *SAT1* *SH3BGRL* *SHROOM4* *SLC10A3* *SLC38A5* *SYTL4* *THOC2* *TIMP1* *TMSB4X* *TMSB4Y* *TNS1* *TRIM26* *UBL4A* *UPF3B* *VDR* *WDR13* *WDR44* *XK*	*COX11* *HLA-DRB1* *IL2RG* *KLHL3* *MAGEE1* *OGT* *PITRM1* *PRKX* *REPIN1* *RPL10* *STK26* *TGFBR3* *UBA1* *VMA21* *ZMYM3*
Non-anorexic cancer patients vs. controls	*CDK2AP1* *NCOR2* *NLGN4Y* *RYR2* *SLC1A3*		*ASAP2* *ATL1* *CETP* *CYFIP1*		
Anorexic vs. non-anorexic cancer patients		*MAST2*	*SLC2A1-AS1*		

## Data Availability

The data that support the findings of this study are not openly available due to reasons of sensitivity and are available from the corresponding author upon reasonable request.

## Supplementary Materials

The following supporting information can be downloaded at https://www.mdpi.com/article/10.3390/nu16213721/s1. Table S1: Genes selected for DNA methylation validation and primer sequences for targeted bisulfite sequencing; Table S2: Methylation differences in selected CGs in anorexic cancer patients compared with controls, obtained from both methods. Table S3: Most enriched pathways of the hypomethylated genes in the comparison between anorexic cancer patients and healthy controls.; Table S4: Most enriched pathways of hypermethylated genes in the comparison between anorexic cancer patients and healthy controls.
