# Supplementary material for: DNA Methylation Signatures Characterize Gene Expression Modulation in Lung Cancer Patients Affected by Anorexia
Source: Nutrients. 2024 Oct 31;16(21):3721. doi: 10.3390/nu16213721 (PMC11547925; doi:10.3390/nu16213721)
Supplement: Supplementary file 1 [file nutrients-16-03721-s001.zip › nutrients-3255167-supplementary.pdf]

Supplementary materials

Supplementary Table S1. Genes selected for DNA methylation validation and primer sequences for targeted bisulfite sequencing

Gene	chr	Amplicons			Primer Sequences	#CGs
		bp	Start	End		
FHL1	chrX	102	135228944	135229046	F: GGGTTTAGTAAATTGAATGTTGAGTGAATG<br>R: ACCCCTCCCCTTAATAACAAAC	8
		176	135229236	135229412	F: GTGTTTTYGTTATTGGAGAGGGT<br>R: CACTTAACTATCTTTATTACTCCCACCTC	21
		15	135229185	135229200	F: GAGTAATAAAGATAGTTAAGTGAGGGTGG<br>R: TCAAAAAATACRCAAATTAACRAAAACCC	3
GNL3L	chrX	195	54556362	54556557	F: ATTGTTTGGTTATTGAGATTTGGGAG<br>R: AACRAAACTTCTAACTAACAACTCAC	18
		121	54556580	54556701	F: GGGATTTGAGAAGYGAGGGTAATA<br>R: AACRAACRTAAACTATCAATCAAAAACTCC	17
PGRMC1	chrX	213	118370042	118370255	F: GAGGTTGGGTTGGGGTTTTTTA<br>R: CCACTTTCTCCTCCCTCTAAAC	26
FLNA	chrX	158	153603114	153603272	F: AGAAAGTTTTAATTGGTAAAATTGTTTAGGAGTT<br>R: ACACRAACCCTACAATCCCTAT	22
		226	153598677	153598903	F: GGTATTGGGTAGGTTTTTGGTTTTG<br>R: ACAATTTTACTAACTATACTTCTTTCTAACCCC	8

Supplementary Table S2. Methylation differences of selected CGs in anorexic cancer patients compared to controls, obtained from both methods.

		Microarray*	Targeted bisulfite sequencing[§]
Gene	CG position	delta beta	methylation difference
GNL3L	chrX:54556391	-0.3	-0.6
	chrX:54556443	-0.3	-0.6
	chrX:54556446	-0.4	-0.6
	chrX:54556669	-0.3	-0.2
	chrX:54556671	-0.3	-0.3
	chrX:54556693	-0.3	-0.2
FHL1	chrX:135228969	-0.2	-0.2
	chrX:135229045	-0.3	-0.2
	chrX:135229187	-0.2	-0.3
	chrX:135229363	-0.2	-0.3
	chrX:135229383	-0.2	-0.2
FLNA	chrX:153598743	-0.3	-0.3
	chrX:153598903	-0.2	-0.3
	chrX:153603126	-0.2	-0.2
	chrX:153603154	-0.2	-0.3
	chrX:153603171	-0.3	-0.2
PGRMC1	chrX:118370063	-0.3	-0.1
	chrX:118370148	-0.2	-0.1
	chrX:118370162	-0.3	-0.1

*In microarray analysis, delta beta is the average beta value of anorexic cancer patients minus average beta value of healthy controls and p-value is calculated by GenomeStudio software by Illumina. A delta beta >0.2 or <-0.2 is used to identify respectively hypomethylated or hypermethylated genes.

[§]In targeted bisulfite sequencing, methylation difference is the difference in methylation ratios between anorexic cancer patients and healthy controls and p-value is calculated performing fisher's exact test. A methylation difference <-0.1 or >0.1 is used to identify respectively hypomethylated or hypermethylated genes.

All the methylation differences reported in table and obtained with both methods showed p-values <0.0001.

Supplementary Table S3. Most enriched pathways of the hypomethylated genes in the comparison between anorexic cancer patients and healthy controls.

Panther pathways	P-value	Adjusted P-value	Genes
PDGF signaling pathway Homo sapiens P00047	4,50E+11	0.031078	RPS6KA3;RPS6KA6;OPHN1;ELF4;ARAF;MAPK1;ELK1;ARHGAP6;ARHGAP4;VAV2;RAB11B
Insulin/IGF pathway-mitogen activated protein kinase kinase/MAP kinase cascade Homo sapiens P00032	0.001407	0.048546	RPS6KA3;RPS6KA6;IRS4;MAPK1;ELK1
Interleukin signaling pathway Homo sapiens P00036	0.013098	0.268390	RPS6KA3;RPS6KA6;IL10RB;ARAF;MAPK1;ELK1;IL13RA1
Ras Pathway Homo sapiens P04393	0.015558	0.268390	RPS6KA3;RPS6KA6;ARAF;MAPK1;PAK3;ELK1
Histamine H2 receptor mediated signaling pathway Homo sapiens P04386	0.026914	0.371423	PRKACG;PRKX
5HT2 type receptor mediated signaling pathway Homo sapiens P04374	0.045206	0.401313	PRKCH;HTR2C;CACNA1F;PRKCZ
Beta2 adrenergic receptor signaling pathway Homo sapiens P04378	0.04765818744317723	0.40131377058621437	PRKACG;PRKX;CACNA1F
Beta1 adrenergic receptor signaling pathway Homo sapiens P04377	0.04765818744317723	0.40131377058621437	PRKACG;PRKX;CACNA1F
T cell activation Homo sapiens P00053	0.06285756557605351	0.40131377058621437	ARAF;WAS;MAPK1;PAK3;VAV2
Dopamine receptor mediated signaling pathway Homo sapiens P05912	0.06555652829193867	0.40131377058621437	MAOA;PRKACG;FLNA;PRKX

Supplementary Table S4. Most enriched pathways of hypermethylated genes in the comparison between anorexic cancer patients and healthy controls.

Pather pathways	P-value	Adjusted P-value	Genes
FGF signaling pathway Homo sapiens P00021	0.055411	0.524542	MAP2K3;PRKCA;FGF11
Toll receptor signaling pathway Homo sapiens P00054	0.067838	0.524542	MAP2K3;TAB1
EGF receptor signaling pathway Homo sapiens P00018	0.069735	0.524542	MAP2K3;ERBB4;PRKCA
Endothelin signaling pathway Homo sapiens P00019	0.138724	0.524542	GNAS;PRKCA
Integrin signalling pathway Homo sapiens P00034	0.154978	0.524542	MAP2K3;ARPC3;ITGA1
Endogenous cannabinoid signaling Homo sapiens P05730	0.182158	0.524542	CACNA1G
Alpha adrenergic receptor signaling pathway Homo sapiens P00002	0.182158	0.524542	PRKCA
Oxidative stress response Homo sapiens P00046	0.189281	0.524542	MAP2K3
Histamine H1 receptor mediated signaling pathway Homo sapiens P04385	0.203343	0.524542	PRKCA
Beta2 adrenergic receptor signaling pathway Homo sapiens P04378	0.217163	0.524542	RYR2
